# BMAA, Methylmercury, and Mechanisms of Neurodegeneration in Dolphins: A Natural Model of Toxin Exposure

**DOI:** 10.3390/toxins13100697

**Published:** 2021-10-01

**Authors:** David A. Davis, Susanna P. Garamszegi, Sandra Anne Banack, Patrick D. Dooley, Thomas M. Coyne, Dylan W. McLean, David S. Rotstein, Deborah C. Mash, Paul Alan Cox

**Affiliations:** 1 Department of Neurology, Miller School of Medicine, University of Miami, Miami, FL 33136, USA; sgaramszegi@med.miami.edu (S.P.G.); pdd29@miami.edu (P.D.D.); dwill7@bu.edu (D.W.M.); dmash@med.miami.edu (D.C.M.); 2 Brain Chemistry Labs, Institute for Ethnomedicine, Jackson, WY 83001, USA; sandra@ethnomedicine.org (S.A.B.); paul@ethnomedicine.org (P.A.C.); 3 Office of the District 21 Medical Examiner, Fort Myers, FL 33907, USA; tmcoynemdphd@gmail.com; 4 Marine Mammal Pathology Services, Olney, MD 20832, USA; drdrot@gmail.com; 5 Department of Molecular and Cellular Pharmacology, Miller School of Medicine, University of Miami, Miami, FL 33136, USA; 6 Dr. Kiran C. Patel College of Allopathic Medicine, Nova Southeastern University, Davie, FL 33328, USA

**Keywords:** Alzheimer’s disease, animal models, anthropogenic contaminates, blue-green algae, cetaceans, cyanotoxins, neurofibrillary tangles, TDP-43

## Abstract

Dolphins are well-regarded sentinels for toxin exposure and can bioaccumulate a cyanotoxin called β-*N*-methylamino-l-alanine (BMAA) that has been linked to human neurodegenerative disease. The same dolphins also possessed hallmarks of Alzheimer’s disease (AD), suggesting a possible association between toxin exposure and neuropathology. However, the mechanisms of neurodegeneration in dolphins and the impact cyanotoxins have on these processes are unknown. Here, we evaluate BMAA exposure by investigating transcription signatures using PCR for dolphin genes homologous to those implicated in AD and related dementias: *APP*, *PSEN1*, *PSEN2*, *MAPT*, *GRN*, *TARDBP*, and *C9orf72*. Immunohistochemistry and Sevier Münger silver staining were used to validate neuropathology. Methylmercury (MeHg), a synergistic neurotoxicant with BMAA, was also measured using PT-GC-AFS. We report that dolphins have up to a three-fold increase in gene transcription related to Aβ^+^ plaques, neurofibrillary tangles, neuritic plaques, and TDP-43^+^ intracytoplasmic inclusions. The upregulation of gene transcription in our dolphin cohort paralleled increasing BMAA concentration. In addition, dolphins with BMAA exposures equivalent to those reported in AD patients displayed up to a 14-fold increase in AD-type neuropathology. MeHg was detected (0.16–0.41 μg/g) and toxicity associated with exposure was also observed in the brain. These results demonstrate that dolphins develop neuropathology associated with AD and exposure to BMAA and MeHg may augment these processes.

## 1. Introduction

Dolphins are exposed to a number of environmental stressors that can alter behavior and reduce lifespan [1,2,3]. A common insult faced by dolphins are environmental toxins, which are linked to a number of mortality events [4,5]. One emerging toxin is β-*N*-methylamino-l-alanine (BMAA), a non-protein amino acid produced by cyanobacteria that has been linked to neurodegenerative disease, including Alzheimer’s disease (AD) and amyotrophic lateral sclerosis (ALS) [6,7,8]. BMAA can biomagnify in marine and terrestrial food chains, where it can bioaccumulate in marine apex predators and humans [6,8,9,10,11,12]. Exposure to BMAA causes excitotoxicity in neurons, glial activation, and tangled proteins in the brain [13,14]. Our laboratory detected BMAA in brains of stranded dolphins at concentrations higher than those found in individuals with AD and ALS [8,15]. Furthermore, the same dolphins possessed concurrent pathological hallmarks of AD, suggesting cyanotoxin exposure could be associated with the occurrence or the progression of neuropathology [15,16,17].

In addition to cyanotoxins, anthropogenic contaminates (ACs) generated by agricultural and industrial processes can persist in the aquatic environment, are poorly metabolized, and can cause toxicity. ACs are potential threats to dolphins and other marine mammals [18]. ACs such as methylmercury (MeHg), a potent neurotoxicant, can also biomagnify in the marine food web and concentrate in the brain [19]. Therefore, the combined exposures to both cyanotoxins and ACs are a major concern [20]. For example, in vitro, co-application of MeHg and BMAA caused synergistic necrosis to neurons [21]. Thus, experimental models are needed to better understand the potential neurotoxic interactions between cyanotoxins and ACs.

Dolphins provide a non-transgenic and natural model of toxin exposure [17,22]. Understanding the progression of toxin-related neurodegeneration in this marine mammal would provide relevant information regarding potential human exposures [22]. Here, we performed gene expression and histopathological analysis on the brains of dolphins with documented exposure to the BMAA toxin [15]. Gene transcription markers related to common neurodegenerative diseases in humans were evaluated in dolphin brain regions involved in cognitive and motor functions with qPCR primers specific to the dolphin genome. Neuropathological analysis was also performed on the same cohort. In addition, MeHg was measured in brain tissues to evaluate a potential co-exposure with BMAA.

## 2. Results

### 2.1. Stranded Dolphins

Short-beaked common dolphins (*Delphinus delphis*; *n* = 7) observed stranded in Massachusetts between the months of March and April in 2012 that were examined by Davis et al. were assessed in this study. The remaining dolphins analyzed by Davis et al. could not be included in our analyses due to the exhaustion of these specimens [15]. Our dolphin cohort consisted of 43% females and 57% males, among which 43% were adults and 57% were subadults. Necropsy findings ranged from brucellosis infection to unknown causes of death. The median and interquartile range (IQR) weight of dolphins in our cohort was 77.0 (45.0) kg. The median length was 185 (39) cm and the average RIN value from RNA extracted from the dolphin brain was 9.6 (0.60) (Table S1).

### 2.2. BMAA Exposure

**Figure 1:**
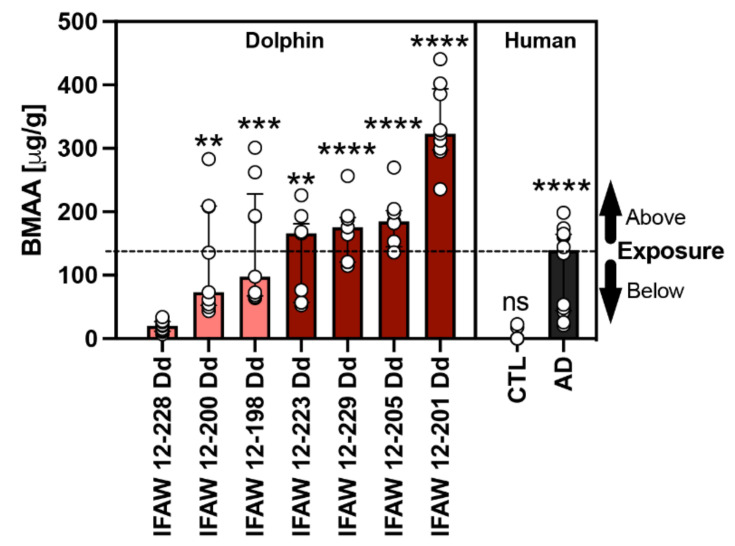
Comparative BMAA Toxicology. The BMAA toxin was detected in the parietal lobe region of stranded dolphins (*n* = 7) [15] and the cerebral cortex of human postmortem brain samples from non-demented (CTL; *n* = 12) and Alzheimer’s disease (AD) patients (*n* = 12) using HPLC-FD [8]. The median BMAA concentration detected was 165.9 (112.0) μg/g with concentrations ranging from 20.2 to 323.3 μg/g across all dolphins. Each dolphin was ranked based on their BMAA concentrations and then compared with the levels of BMAA detected in humans with clinically diagnosed AD (139.5 (120.6) μg/g). Using this comparison, our dolphin cohort was then divided into two categories based on environmental BMAA exposure concentration being less than (salmon bars) or greater (cayenne bars) than those found in AD patients (black bar). Dolphin analysis (**, *p* = 0.0027, ***, *p* = 0.0002; **, *p* = 0.0016; ****, *p* < 0.0001; ANOVA); Human analysis: (****, *p* < 0.0001; ns, no significance Mann Whitney Test).

BMAA and its structural isomers (2,4-DAB & AEG) were previously detected in our dolphin cohort and reported by Davis et al. in 2019 [15]. The median concentration of BMAA detected across all dolphins was 166.0 (112.0) μg/g and ranged from 20.2 to 323.3 μg/g. To determine the relationship of toxin exposure to gene transcription signatures, we ranked dolphins by BMAA exposure (Table 1). For gene transcription and neuropathology assessments, dolphins were grouped in the following two categories based on their environmental exposure (EE) to BMAA either being less than ([EE] < AD; 20.2–97.7 μg/g; *n* = 3) or greater than ([EE] > AD; 166.0–323.3 μg/g; *n* = 4) the concentration found in patients diagnosed with AD (139.5 (120.6) μg/g; *n* = 12) (Figure 1) [8]. To normalize our PCR data, we used dolphin IFAW 12-228 Dd, which had the lowest detectable BMAA concentration and was relatively free of AD neuropathology (Figure 1).

### 2.3. Gene Expression Markers

**Figure 2:**
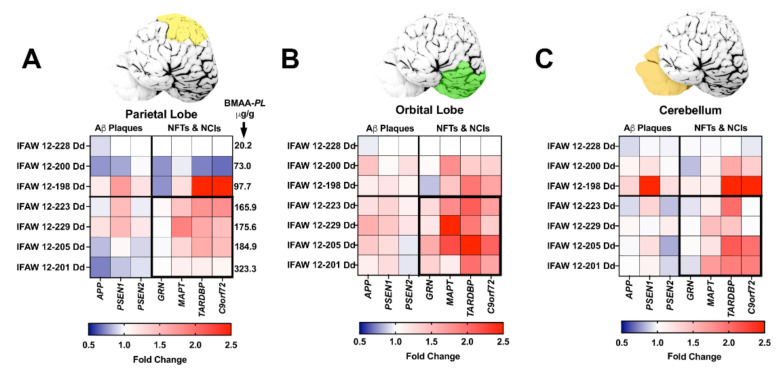
Brain Region-Specific Analysis of Gene Transcription. (**A**–**C**) qPCR was performed to determine the fold change in transcription levels of genes involved in the development of amyloid-beta (Aβ^+^) plaques, neurofibrillary tangles (NFTs), and neuronal intracytoplasmic inclusions (NCIs). Top panels: Tissue samples were taken from the parietal lobes (*PL*, yellow), orbital lobe (*OrL*, green), and cerebellum (*Cer,* orange). Bottom panels: Heat maps displaying the relative fold change in gene expression of genes analyzed ranked in order of increasing BMAA exposure. Dolphin IFAW 12-228 Dd was used as a normalization control. BMAA concentrations measured in the *PL* region is indicated in panel A. Dolphins displayed upregulated gene transcription for all seven genes across all three brain regions. The *OrL* region showed the most upregulated transcription of genes, especially in genes involved in development of NFTs and NCIs (B). Gene transcription accounted for 24.6% of the total variance in the *PL*, 41.4% in the *OrL* and 34.3% in the *Cer*. Whereas, BMAA exposure accounted for 37.8% of the total variance in the *PL*, 26.4% in the *OrL*, and 28.9% in the *Cer* (*p* < 0.0001 Two Way ANOVA).

**Figure 3:**
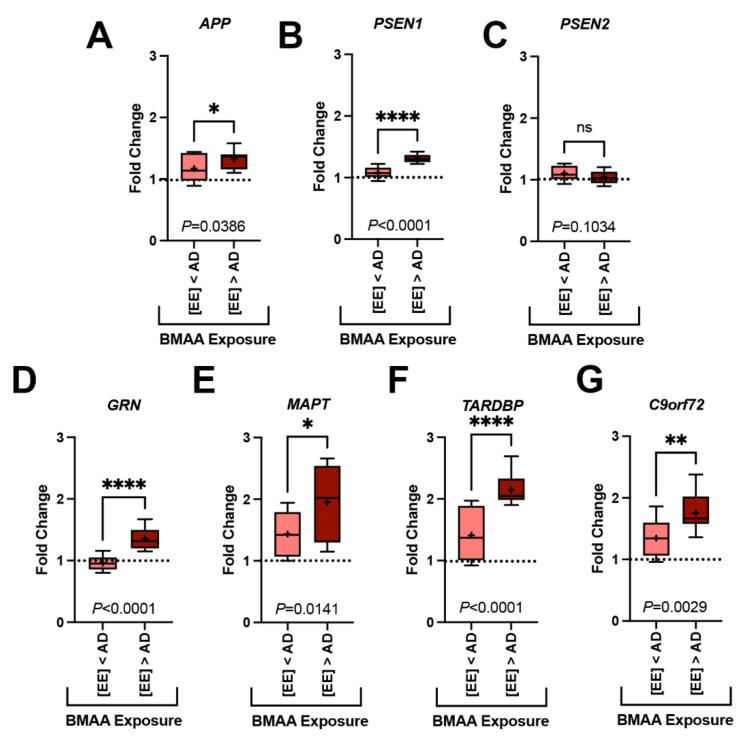
Gene Transcription and BMAA Exposure. Dolphins were categorized into groups based on their BMAA exposure being less than ([EE] < AD) or greater than ([EE] > AD) those concentrations observed in AD patients. Gene expression markers measured in the orbital lobe region are shown above. Dotted line indicates the fold change of our dolphin with the lowest BMAA exposure (IFAW 12-228 Dd). (**A**–**C**) Transcription of genes implicated in the development of amyloid-beta plaques were modestly increased up to 1.2-fold in [EE] > AD dolphins (*APP*, *, *p* = 0.0386; *PSEN1*, ****, *p* < 0.0001; ns, *PSEN2*, *p* = 0.1034; *t*-Test). (**D**–**G**) Whereas, genes responsible for the development of neurofibrillary tangles and neuronal intracytoplasmic inclusions had a more robust increase in transcription (1.5-fold) (*GRN*, **** *p* < 0.0001; *MAPT*, * *p* = 0.0141; *TARDBP*, **** *p* < 0.0001; *C9orf72*, ** *p* = 0.0029, *t*-Test) (ns: ns, no significance).

All seven genes of interest were expressed in the parietal lobe (*PL*), orbital lobe (*OrL*), and cerebellum (*Cer*) brain regions (Supplementary Table S2, Figure 2). Each gene displayed a region-specific expression (Figure 2A–C). The median fold changes in expression for six of seven gene markers were upregulated above baseline (Supplementary Table S3). Transcription levels were most robust in the *OrL* brain region, especially for the APP (*p* = 0.0187, ANOVA) and MAPT (*p* = 0.0460, ANOVA) genes (Supplementary Table S3, Figure 2B). In addition, dolphins with the highest fold change in gene transcription also had the highest BMAA exposure (Figure 3). The increase in gene expression was especially observed in the MAPT (*p* = 0.0141; ANOVA) and TARDBP (*p* < 0.0001; ANOVA) genes, which were upregulated in some dolphins as high as 2.6- and 2.9-fold above baseline, respectively (Figure 3E,F and Supplementary Table S3).

### 2.4. Neurofibrillary Tangles

**Figure 4:**
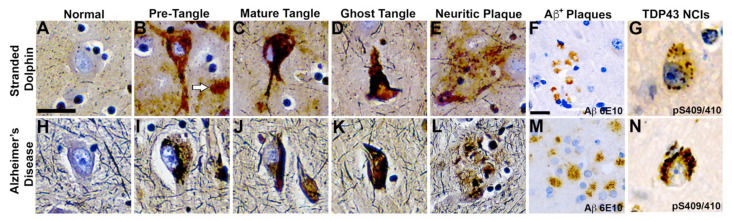
Comparative Neurohistopathology. Sevier Münger silver staining highlights neurofibrillary tangles (NFTs), neuritic plaques (NPs), neurointracytoplasmic inclusions (NCIs), and amyloid-beta (Aβ^+^) plaque morphology in the dolphin brain that are analogous to those found in advanced Alzheimer’s disease (AD). (**A**,**H**) Intact neurons with normal cytoarchitecture. (**B**,**I**) Early NFTs with granular inclusions surrounding the cell soma and processes. Arrow highlights an adjacent diffused plaque. (**C**,**J**) Mature NFTs with dense paired helical filaments and nuclear changes indicative of dying neurons. (**D**,**K**) Ghost or tombstone tangles characterizing dead neurons. (**E**,**L**) NPs containing dense cores surrounded by neuronal cell bodies and processes. (**F**,**M**) Clusters of Aβ^+^ plaques and (**G**,**N**) pathological TAR DNA-binding protein 43 (TDP-43) NCIs in the orbital lobe region of dolphins and the frontal cortex of an 84-year-old female with advanced AD. Scale bars = 25 μm.

**Figure 5:**
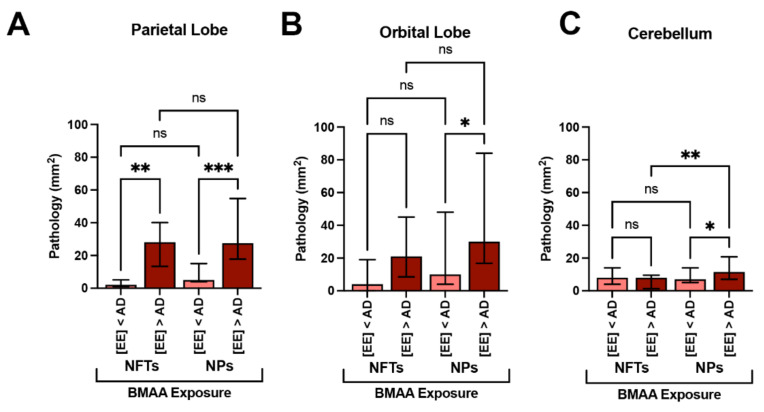
Alzheimer’s Disease Neuropathology and BMAA Exposure. (**A**) In the parietal lobe, dolphins with BMAA exposure equivalent or greater than those found in Alzheimer disease (AD) patients ([EE] > AD group, *n* = 20 tissue sections from 4 dolphins) showed a 14-fold increase in neurofibrillary tangles (NFTs) and a 5.2-fold increase in neuritic plaques (NPs) in comparison to dolphins with less exposure ([EE] < AD; *n* = 15 tissue sections from 3 dolphins) (**, *p* = 0.0013; ***, *p* = 0.0001; ANOVA). (**B**) The orbital lobe, also showed a 5.5- and 3.2-fold increase in NFTs and NPs, respectively (*, *p* = 0.0196; ANOVA; ns = no significance). (**C**) NFT neuropathology in the cerebellum was relatively unchanged with BMAA exposure. However, the density of NPs increased 1.6-fold in the [EE] > AD (**, *p* = 0.0356; **, *p* = 0.0052; ns = no significance).

We have previously demonstrated that short-beaked common dolphins develop dystrophic neurites and neuropil threads in the auditory and visual areas of the cerebral cortex [15]. Here, we show that the same dolphins possessed NFTs, a hallmark of AD, in the PL, OrL, and Cer brain regions (Figure 4). We observed numerous and widespread NFTs with morphological characteristics similar to those found in advanced AD (Figure 4A–E,H–L). Quantitative image analysis of brain sections shows that the *PL* region had the greatest median density of NFTs at 26.0 (30.0) per mm^2^ across all stranded dolphins. A similar density of NFTs was observed in the *OrL* region, at 15.0 (23.0) per mm^2^, and the *Cer* region had the lowest density of NFTs 8.0 (3.0) per mm^2^. Dolphins with BMAA exposures equivalent to or above those found in AD patients ([EE] > AD group) had a 14.0-fold increase in the density of NFTs in the *PL* (*p* = 0.0013, two-way ANOVA) and 5.2-fold increase in the *OrL* (ns, Two-Way ANOVA) (Figure 5A,B). The density of NFTs in the *Cer* region remained unchanged regardless of BMAA exposure (Figure 5C).

### 2.5. Neuritic Plaques

We have previously shown that dolphins developed widespread Aβ^+^ plaques in the cerebral cortex and brainstem [15]. Here, we show the same dolphins possess Aβ^+^ plaques in the *PL*, *OrL*, and *Cer* brain regions and are associated with NPs, a form of plaque most correlated with dementia (Figure 4E,F) [23]. The density and morphology of these lesions were highly similar to those observed in AD (Figure 4L,M). NPs, like NFTs, displayed a similar density and distribution across our brain regions of analysis. The *PL* region had the greatest median density at 23.0 (29.0) per mm^2^ followed by the *OrL* region, at 19.0 (48.0) per mm^2^. Again, the *Cer* region had the lowest density of NPs 13.0 (10.0) per mm^2^. The [EE] > AD dolphin group had a 5.5-fold greater density of NPs in the *PL* (*p* = 0.0013, two-way ANOVA) and 3.0-fold density in the *OrL* (ns, two-way ANOVA) (Figure 5A,B). However, unlike NFTs, we observed a 1.6-fold increase in NPs in the *Cer* region in the [EE] > AD group (*p* = 0.0356, two-way ANOVA) (Figure 5C).

### 2.6. TDP-43 Neuronal Intracytoplasmic Inclusions

The pathological form of phosphorylated TDP-43, a protein encoded by the *TARDBP* gene (Figure 3F), was also observed in the dolphin brain (Figure 4G). All dolphins displayed widespread TDP-43 NCIs regardless of their BMAA exposure category. TDP-43 NCIs were observed throughout all cortical layers and had similar morphological characteristics to those observed in AD patients (Figure 4G,N).

### 2.7. Methylmercury Exposure

**Figure 6:**
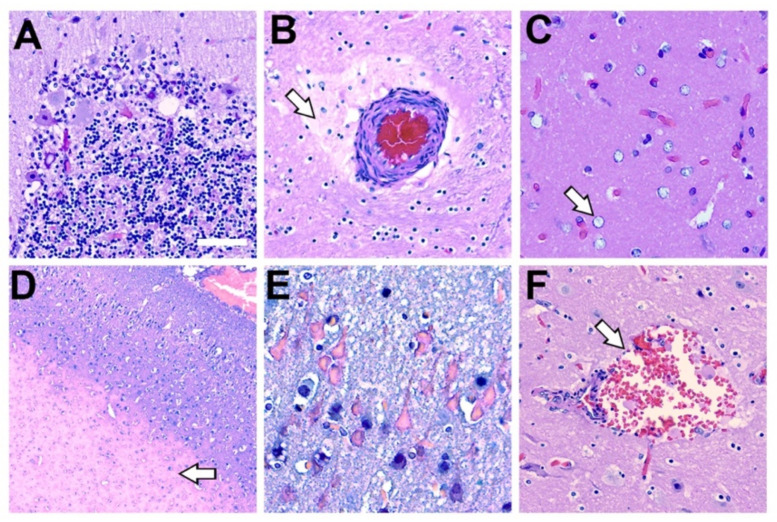
Histopathology Associated with Methylmercury Toxicity and Dolphin Stranding. (**A**) Cerebellar Purkinje neurons display disorganization with pallor of the perikaryon (chromatolysis) and vacuolation. In addition, granular cell loss and microcavitation with accompanying gliosis was observed in the cerebellum of dolphin IFAW 12-228 Dd. (**B**) A blood vessel that displayed thickening of the adventitia and media with expansion of the perivascular spaces, indicative of continuous seepage of serum proteins was observed in IFAW12-228 Dd. (**C**) Alzheimer’s type-2 astrocytes (arrow), a cellular marker associated with toxin exposure, was observed in the orbital lobe of dolphin IFAW 12-205 Dd. (**D**) Microvascular lesion in the parietal lobe. The microinfarct has a large area of subcortical necrosis (arrow), with hypoxic-ischemic neurons in the superficial cortical layers of dolphin IFAW 12-198 Dd (**E**). (**F**) Subarachnoid hemorrhage observed in the *OrL* region containing erythrocytes and activated macrophages in Virchow-Robin space (arrow) in dolphin IFAW 12-228 Dd. (40× digital pathology scan; Scale bar = 250 μm).

MeHg was detected in the *PL* region of all dolphins (seven in seven; 100%). The median concentration of MeHg was 0.278 (0.23) μg/g and ranged from 0.163–0.411 μg/g (Table 1). The ratio of tissue BMAA to MeHg was 527:1 (430.0) and ranged from 49:1–1121:1. Pathological changes commonly associated with MeHg neurotoxicity were also present. We observed neuronal atrophy and loss of the cerebellum, neuropil rarefaction, gliosis, and microinfarcts across all MeHg^+^ dolphins in our study cohort (Figure 6).

### 2.8. Additional Histopathological Findings

Our dolphin cohort also displayed several neurohistopathological changes that are commonly observed in stranded cetaceans. We observed wide-spread hypoxic ischemic changes in neurons of the cerebral cortex and cerebellum (Figure 6E). In addition, we observed vascular changes such as hemorrhage in the perivascular space with apparent protein leakage, which may be part of a terminal process associated with cardiovascular collapse (Figure 6B,F). Finally, gross and microscopic evaluation for pathological changes associated with neurobrucellosis were not observed (Supplementary Figure S1).

## 3. Discussion

Links between chronic dietary exposure to environmental toxins and progressive neurodegenerative disease continue to accumulate [24,25,26,27,28,29]. Here, we investigate the effects of BMAA and MeHg, two environmental neurotoxins known to bioaccumulate in the marine food chain, concentrate in apex predators, and have synergistic effects on neural cells [19,30,31,32,33]. Our necropsy cohort consisted of a small group of dolphins that were found stranded in Massachusetts, which frequently reports a number of harmful algal blooms and MeHg contamination [4,34,35]. Our study aimed to detect BMAA and MeHg in brain tissues, determine if the bioaccumulation of these neurotoxins could induce or potentiate neurodegenerative changes, and relate these findings to potential human exposures [36,37].

Here, we show both BMAA and MeHg present in the brains of stranded dolphins. The occurrence of both neurotoxins in CNS tissues may suggest a potential mechanism for synergies through chronic dietary exposure [38,39]. When absorbed into target tissues, the elimination half-life of these toxins can range from 1 to 120 days [40,41,42,43]. Thus, the slow removal of these molecules from the brain provides a toxic reservoir that can cause neuronal injury over the course of years [11]. BMAA is a nonprotein amino acid that crosses the blood brain barrier (BBB), where it can enter the free amino-acid pool and incorporate into proteins at serine residues to cause misfolding [14,40,44,45]. Chronic dietary exposure to BMAA has been linked to ALS/ Parkinson dementia complex (PDC) of Guam and causes a neurodegenerative phenotype associated with AD and ALS in primate models [14,45,46,47,48]. Since BMAA has been documented in diverse ecosystems around the world, exposure of marine and terrestrial mammals to this toxin is a global concern [38].

In addition to toxin exposures, we also demonstrate the same dolphins displayed increased expression of gene markers and neuropathology associated with the onset of AD and related dementias (ADRD). Both the gene expression and the severity of neuropathology were amplified in dolphins with BMAA exposures equivalent to those reported in AD patients [6,8]. TDP-43 NCIs, which have been implicated in AD, ALS/PDC, and several other dementias were also observed in the dolphin cerebral cortex and cerebellum [49,50,51,52,53,54]. Concurrent TDP-43 proteinopathy is associated with greater atrophy of the hippocampus and accelerated cognitive decline in humans [52]. Here, we show that stranded dolphins positive for the BMAA toxin have increased *TARDBP* gene transcription and frequent TDP-43 protein intracytoplasmic inclusions. The abnormal regulation of the TDP-43 gene and protein provides a potential mechanism of synergy for BMAA and MeHg [13,45,55,56,57]. The presence of AD-like pathology with concurrent TDP-43 lesions further supports the use of dolphins as a natural model of neurodegenerative disease [15,16,17]. Future studies are needed to understand the impact of toxin exposure on *TARDBP* gene transcription and the occurrence of TDP-43 proteinopathy in short-beak common dolphins.

MeHg is a neurotoxic organometallic cation form from inorganic mercury in the marine environment [58]. The most predominant route of MeHg exposure is the consumption of contaminated fish and seafood [12,33,58]. Once ingested, MeHg is absorbed and transported freely across the BBB where it can strongly associate with thiol groups on proteins to cause oxidative stress, disrupt calcium homeostasis, and trigger neuronal death [59]. MeHg exposure has been implicated in Minamata disease, a crippling neurological disorder affecting more than 2200 patients that consumed contaminated fish and seafood from Minamata Bay in Japan between the 1950s and 1960s [60]. Governments have now provided strict regulation and guidelines on the recommended weekly intake of MeHg [61]. However, there is a concern that long term subclinical doses of MeHg can cause cognitive impairment, chronic disease, and has been proposed to be a possible contributor to the onset of AD [33,62,63,64].

In our study cohort, the levels of MeHg detected were in the range of those found in autopsy specimens of humans with chronic low dose poisoning [65,66,67]. Moreover, we observed neurotoxic changes associated with MeHg exposure, including neuronal loss and gliosis in the cerebellum [30]. However, due to our small sample size and the significant overlap between neuropathology and MeHg neurotoxicity, it was difficult to determine MeHg’s synergy with BMAA. However, the presence of both toxins suggests a synergistic or, at minimum, additive potential to induce neurodegeneration. Possible mechanisms of combined toxicity may include both BMAA’s and MeHg’s ability to cause glutathione depletion, glutamate dyshomeostasis, mitochondrial dysfunction, and the stimulation of the unfolded protein response [15,44,68,69,70,71].

Finally, dolphins can develop infectious diseases that can cause behavioral changes, neurodegeneration, and mortality events [2,72,73]. In our cohort, three dolphins were diagnosed with brucellosis, a bacterial infection common to marine and terrestrial mammals as well as humans [74]. The *Brucella ceti* bacteria can cause chronic illnesses ranging from skin lesions to neurobrucellosis [74]. In this study, gross and microscopic findings associated with *B*. *ceti* neuroinfection in our dolphins with brucellosis were not observed [75,76]. In addition, the *B*. *ceti* infection did not have an effect on ADRD gene transcription nor the severity of neuropathology. However, the presence of neuroinfection and inflammation should be considered when assessing neuropathological changes from cyanotoxin or MeHg exposure, as their effects can contribute to the progression of neurodegeneration.

## 4. Conclusions

We demonstrate that stranded dolphins demonstrate an upregulated transcription of genes linked to human neurodegenerative diseases. Dolphins also possessed AD-type pathological changes. The progression of AD pathology was paralleled by increasing BMAA exposure. Furthermore, dolphins displayed pathological TDP-43 inclusions and MeHg neurotoxicity, both known to modify the course of dementia in humans. Taken together, this dolphin model further supports that environmental exposure over the lifespan may represent a risk factor for developing neurodegenerative disease.

## 5. Methods and Materials

### 5.1. Dolphins

Female and male short-beaked common dolphins subadult to adult (*n* = 7; *Delphinus delphis*) were collected from stranding sites in Massachusetts in 2012 under a federal permit authorized by the National Ocean and Atmospheric Administration (NOAA). The estimated age class for dolphins was determined as described in Geraci et al. [77]. Physical assessments were performed on-site and dolphins in poor health were euthanized. No dolphin was euthanized for the purpose of this research study. Necropsies were performed within 24–48 h by the Woods Hole Oceanographic Institute Marine Research Facility (WHOI MRF) and International Fund for Animal Welfare (IFAW) [77]. Gross assessments were performed with ancillary pathogen testing where appropriate (Supplementary Table S1). One brain hemisphere was frozen at −80 °C and the contralateral preserved in 10% buffered formalin. Hemispheres were alternated to randomize laterality. Frozen parietal lobe (*PL*), orbital lobe (*OrL*), and cerebellum (*Cer*) were sampled for PCR and histopathology assays.

### 5.2. Extraction of Dolphin RNAs

Total ribonucleic acid (RNA) was extracted from 100 mg of frozen tissue sampled from the *PL*, *OrL* and *Cer* regions using RNeasy Lipid Tissue Mini Kit (Qiagen Inc., Germantown, MD, USA). DNase I on-column treatment (Qiagen Inc., Germantown, MD, USA) was applied to samples to eliminate genomic DNA. RNA concentrations were measured for each sample using a NanoDrop 2000 Spectrophotometer (Thermo Fisher Scientific, Waltham, MA, USA). To determine the quality of RNA, Agilent 2100 Bioanalyzer (Agilent Technologies Inc., Santa Clara, CA, USA) was used to obtain an RNA integrity number (RIN) (Supplementary Table S1). To perform our gene expression analysis, 5 μg of total RNA was used to generate complementary DNA (cDNA) libraries for each sample using a High Capacity Reverse Transcription Kit (Thermo Fisher Scientific, Waltham, MA, USA).

### 5.3. qPCR Analysis

Genes related to amyloid beta (Aβ) plaques, neurofibrillary tangles (NFTs), neuritic plaques (NPs), and nuclear and intracytoplasmic inclusions (NCIs) were analyzed: *APP, PSEN1, PSEN2, MAPT, GRN, TARDBP,* and *C9orf72*. Gene expressions were measured using custom dolphin AD PCR assays with a TaqMan Universal PCR Master Mix on QuantStudio^®^ 6 Flex Real-Time PCR System (Thermo Fisher Scientific, Waltham, MA, USA). Custom TaqMan assays were designed by the Thermo Fisher Bioinformatics Team based on the *T. truncatus* genome turTur1, a closely related cetacean species [78], in combination with a limited sequence of *D. delphis* (ncbi.nlm.gov/bioproject/421547; accessed on 27 August 2021) (Supplementary Table S2). As an internal control, gene expression levels were normalized to 40S ribosomal protein S9 (*RPS9*), one of the most stable genes in cetacean species [79]. The average Ct value for *RSP9* sequence was 20.84 ± 0.03 with a coefficient of variation of 1.23% for 63 PCR runs, showing a stable gene expression level. Triplicate samples and a no template control (NTC) were performed for each assay. cDNA (100 ng) was amplified and run at the following conditions: 2 min at 50 °C, 10 min at 95 °C, 40 cycles: 15 sec at 95 °C and 1 min at 60 °C. Data analysis were performed using QuantStudio^®^ 6 Flex Real-Time PCR System Software v1.0. All real-time PCR data files were imported into ExpressionSuite Software v1.0.4 (Applied Biosystems, Waltham, MA, USA) to analyze relative expression across all plates using the comparative Ct method [80]. After normalization of data, a fold change was calculated using a dolphin with the lowest detectable levels of BMAA as a baseline control.

### 5.4. HPLC-FD for BMAA Detection

High-performance liquid chromatography with fluorescence detection (HPLC-FD) measurements previously reported by Davis et al. and Pablo et al. were used in the study [8,15]. Briefly, BMAA was separated from *N*-(2-aminoethyl)glycine (AEG) & 2,4-diaminobutyric acid (2,4-DAB) using reverse-phase elution (Nova-Pak C18 column, 3.9 mm × 300 mm) on a 1525 Binary HPLC pump and a 717 autosampler (Waters Corp., Milford, MA, USA). The mobile phase consisted of Eluent A (140 mM sodium acetate, 5.6 mM triethylamine, pH 5.7) and Eluent B (52% (*v*/*v*) aqueous acetonitrile) using a flow rate of 1.0 mL/min and a 10 μL sample injection volume. Samples were eluted using a 60 min gradient: 0 min 100% A; 2 min 90% A; 5 min 86% A; 10 min 86% A; 18 min 73% A; 30 min 57% A; 35 min 40% A; 37.5 mins100% B; 47.5 min 100% B; 50 min 100% A; 60 min 100% A. The samples were derivatized with an AQC fluorescent tag using 20 μL of sample plus 20 μL AQC in 60 μL of borate buffer. Analytes were separated at 29.6 min (AEG), 31.1 min (BMAA), and 33 min (2,4-DAB). Analyte detection was performed using a 2475 Multi k-Fluorescence Detector (Waters Corp., Milford, MA, USA) with excitation at 250 nm and emission at 395 nm. Measurements (4–5 per dolphin) were compared with those spiked-in controls containing known amounts of standard (L-BMAA HCl, Sigma-Aldrich, Inc. St. Louis, MO, USA). The limit of detection (LOD; 2.7 ng/mL) and limit of quantification (LOQ; 7.0 ng/mL) were based on the standard deviation (SD) of response and slope (S), calculated from the linearity of the response of BMAA. The following formulas were used to obtain the LOD (3.3 × SD)/S and LOQ: (10 × SD)/S. The efficiency of recovery for analytes was estimated by adding known amounts of a BMAA standard spiked into a reference sample below the LOD.

### 5.5. PT-GC-AFS for MeHg Detection

Purge-and-trap gas chromatography-atomic fluorescence spectrometry (PT-GC-AFS) was performed in an laboratory accredited by the National Environmental Laboratory Accreditation Program (NELAP) for the analysis of MeHg [81]. Parietal lobe (*PL*) (200 mg) was mixed with potassium hydroxide (KOH) in MeOH on a heat block, by adding 5 mL of 25% KOH in MeOH solution to the sample, followed by heating on a dry bath for 2 h at 95 °C. After MeHg was extracted into KOH/MeOH solution, MeOH was added into a digestion tube to bring the volume to 10 mL. An aliquot of KOH/MeOH extract (20 μL) was transferred into an amber autosampler vial, which was filled with 30 mL of diH_2_O. Then, 2.0 mL of citric buffer (0.5 M) and 0.05 mL of freshly thawed 1% Sodium tetraethylborate solution was added, immediately after which the vial was topped off with diH_2_O and tightly capped. The vial was put on an autosampler for analysis on a PT-GC-AFS MERX MeHg System (Brooks Rand Instruments, Seattle, WA, USA) where Hg species on the traps were desorbed, separated, pyrolyzed, and detected by AFS. Analytical runs began with an initial calibration containing 5 non-zero points and a system blank. The mean calibration factor (CFm), calculated from the calibration factor (CFx) for Hg in each of the five standards using the system blank-subtracted peak height, was used for the calculation of sample concentration. Each analytical batch included at least one method blank, a continuing calibration check samples (CCS), and a quality control sample (QCS). All method blanks during analysis were below the LOD (0.002 mg/kg). CCS readings were always within acceptable range (85–115% for Hg of initial calibration). Certified reference material, DORM-2, was used as a QCS throughout the analysis and the recoveries for the QCS samples (84–128% for MeHg) were always within acceptable range specified in standard operating procedures (70–130% for MeHg).

### 5.6. Immunohistochemistry

Formalin-fixed paraffin embedded (FFPE) blocks were prepared from dolphin brains as previously described [15]. For the designation of neuroanatomy, we used terminology designated by Oelschlager et al. [82]. The following regions were sampled: parietal lobe (*PL*), orbital lobe (*OrL*), and the cerebellum (*Cer*). Brain tissue sections (5 μm) were prepared for immunohistochemistry as previously described [15]. Slides were stained with hematoxylin and eosin (H&E) and Sevier Münger (SM) silver at AML laboratories using stain kits (American MasterTech, Lodi, CA, USA) [83]. For IHC staining, hydrated slides were incubated in 3% H_2_O_2_ in MeOH for 10 min, followed by rinsing in distilled water for 5 min. Slides were incubated in citrate buffer for 1 h, followed by washing in DiH_2_O on a Thermolyne Roto Mix shaker and incubation in phosphate-buffered saline pH 7.4 (PBS) for 5 min. To block non-specific antibody binding, 10% normal donkey serum (NDS) in PBS was applied to slides in a humidity chamber and incubated at room temperature for 30 min. The following antibodies were applied: anti-β-amyloid 6E10 (1:800, Covance, Ann Arbor, MI, USA), anti-phosphorylated TDP-43 Ser409/410 (1:800; Cosmo-Bio, Inc., Carlsbad, CA, USA). Primary antibodies were incubated overnight at 4 °C. Slides were then rinsed in PBS for 10 min, incubated in 2% NDS for 10 min, then rinsed in PBS. A biotin conjugated goat anti-mouse or rabbit secondary antibody (1:200; Jackson Immunoresearch Laboratories, Inc., West Grove, PA, USA) was incubated on slides for 2 h at room temperature, rinsed with PBS wash for 10 min, followed by the application of Avidin-Biotin Complex (ABC) peroxidase solution (Thermo Fisher Scientific, Waltham, MA, USA) for 1 h. ABC peroxidase was detected using 3,3′-Diaminobenzidine solution (Thermo Fisher Scientific, Waltham, MA, USA) for 10 min. Slides were washed in PBS, rinsed with distilled water, counterstained with Gill No. 1 Hematoxylin, and rinsed with tap water. Brain tissues from an 84-year-old female with advance AD pathological changes was used as a control. The donated tissues were obtained from the University of Miami Brain Endowment Bank, a NIH NeuroBioBank (IRB ethics number, 19920348 (CR00012340)).

### 5.7. Neuropathological Analysis

Histological slides were scanned at 40× (0.2 µm/pixel) using an EasyScan Pro 6 (Motic, Schertz, TX, USA). Scans were annotated and exported to ObjectiveView^TM^ (Objective Pathology, CAN). From each scan, five tiff images (1264 × 704 pixels) were exported to FIJI ImageJ VER2.00-rc-69/1.52p (NIH, Bethesda, MD, USA) for analysis of AD pathology, TDP-43 proteinopathy, and MeHg neurotoxicity. To determine the density of NFTs and NPs, a (2 × 5) grid totaling 1 mm^2^ was applied to cortical layers II and III of the *OrL* and *PL* [84]. The Purkinje cell layer was analyzed for the *Cer*. ImageJ Cell Counter Ver 2.2.2 (University of Sheffield, England, UK) software was used to enumerate pathological lesions.

### 5.8. Statistics

Statistical analyses were performed using Prism Version 9 (Graph Pad, San Diego, CA, USA). Multiple comparisons were analyzed with ANOVA or two-way ANOVA with Dunnett’s or Tukey’s multiple comparison test. Nonparametric data comparisons were conducted using the Mann–Whitney test. The D’Agostino–Pearson and Shapiro–Wilk tests were used to determine normality. Data are presented as the median (interquartile range) and the significance level of alpha = 0.05.

## Figures and Tables

**Table 1:** Toxin Detection and BMAA Exposure Classification of Stranded Dolphins.

Agency ID	Exposure Category	BMAA^ϕ^ (μg/g)	MeHg (μg/g)	BMAA:MeHg
IFAW 12-228 Dd *	[EE] < AD	20.2	0.411	49:1
IFAW 12-200 Dd		73.0	0.163	448:1
IFAW 12-198 Dd		97.7	0.252	388:1
IFAW 12-223 Dd	[EE] > AD	166.0	0.315	527:1
IFAW 12-229 Dd		175.6	0.278	632:1
IFAW 12-205 Dd		185.0	0.165	1121:1
IFAW 12-201 Dd		323.3	0.395	818:1
	**Median (IQR)**	166.0 (112.0)	0.278 (0.230)	572:1 (430.0)
	**Min–Max**	20.2–323.3	0.163–0.411	49:1–1121:1

**^ϕ^** Data derived from Davis et al. 2019. * Dolphin used to normalize PCR gene expression data.

## Data Availability

All data and supporting materials for this study are available within the article and the Supplementary Materials.

## Supplementary Materials

The following are available online at https://www.mdpi.com/article/10.3390/toxins13100697/s1, Supplementary Figure S1: Pathological Findings Associated with Neurobrucellosis Were not Observed in Our Study Cohort; Supplementary Table S1: Stranded Dolphins; Supplementary Table S2: Dolphin qPCR primers; Supplementary Table S3: Gene transcription Fold Change in Stranded Dolphins.

## References

[1] Venn-Watson S.K., Jensen E.D., Smith C.R., Xitco M., Ridgway S.H. (2015). Evaluation of annual survival and mortality rates and longevity of bottlenose dolphins (*Tursiops truncatus*) at the United States Navy Marine Mammal Program from 2004 through 2013. J. Am. Vet. Med Assoc..

[2] Bogomolni A.L., Pugliares K.R., Sharp S.M., Patchett K., Harry C.T., LaRocque J.M., Touhey K.M., Moore M. (2010). Mortality trends of stranded marine mammals on Cape Cod and southeastern Massachusetts, USA, 2000 to 2006. Dis. Aquat. Org..

[3] Jepson P.D., Deaville R., Acevedo-Whitehouse K., Barnett J., Brownlow A., Brownell R.L., Clare F.C., Davison N., Law R.J., Loveridge J. (2013). What caused the UK’s largest common dolphin (*Delphinus delphis*) mass stranding event?. PLoS ONE.

[4] Fire S.E., Bogomolni A., DiGiovanni R.A., Early G., Leighfield T.A., Matassa K., Miller G.A., Moore K.M.T., Moore M., Niemeyer M. (2021). An assessment of temporal, spatial and taxonomic trends in harmful algal toxin exposure in stranded marine mammals from the U.S. New England coast. PLoS ONE.

[5] Danil K., Berman M., Frame E., Preti A., Fire S.E., Leighfield T., Carretta J., Carter M.L., Lefebvre K. (2021). Marine algal toxins and their vectors in southern California cetaceans. Harmful Algae.

[6] Murch S.J., Cox P.A., Banack S.A., Steele J.C., Sacks O.W. (2004). Occurrence of beta-methylamino-l-alanine (BMAA) in ALS/PDC patients from Guam. Acta Neurol. Scand..

[7] Banack S.A., Johnson H.E., Cheng R., Cox P.A. (2007). Production of the neurotoxin BMAA by a marine cyanobacterium. Mar. Drugs.

[8] Pablo J., Banack S.A., Cox P.A., Johnson T.E., Papapetropoulos S., Bradley W.G., Buck A., Mash D.C. (2009). Cyanobacterial neurotoxin BMAA in ALS and Alzheimer’s disease. Acta Neurol. Scand..

[9] Berntzon L., Ronnevi L.O., Bergman B., Eriksson J. (2015). Detection of BMAA in the human central nervous system. Neuroscience.

[10] Brand L.E., Pablo J., Compton A., Hammerschlag N., Mash D.C. (2010). Cyanobacterial blooms and the occurrence of the neurotoxin beta-N-methylamino-L-alanine (BMAA) in South Florida aquatic food webs. Harmful Algae.

[11] Murch S.J., Cox P.A., Banack S.A. (2004). A mechanism for slow release of biomagnified cyanobacterial neurotoxins and neurodegenerative disease in Guam. Proc. Natl. Acad. Sci. USA.

[12] Hammerschlag N., Davis D.A., Mondo K., Seely M.S., Murch S.J., Glover W.B., Divoll T., Evers D.C., Mash D.C. (2016). Cyanobacterial Neurotoxin BMAA and Mercury in Sharks. Toxins.

[13] Yin H.Z., Yu S., Hsu C.I., Liu J., Acab A., Wu R., Tao A., Chiang B.J., Weiss J.H. (2014). Intrathecal infusion of BMAA induces selective motor neuron damage and astrogliosis in the ventral horn of the spinal cord. Exp. Neurol..

[14] Cox P.A., Davis D.A., Mash D.C., Metcalf J.S., Banack S.A. (2016). Dietary exposure to an environmental toxin triggers neurofibrillary tangles and amyloid deposits in the brain. Proc. Biol. Sci..

[15] Davis D.A., Mondo K., Stern E., Annor A.K., Murch S.J., Coyne T.M., Brand L.E., Niemeyer M.E., Sharp S., Bradley W.G. (2019). Cyanobacterial neurotoxin BMAA and brain pathology in stranded dolphins. PLoS ONE.

[16] Gunn-Moore D., Kaidanovich-Beilin O., Gallego Iradi M.C., Gunn-Moore F., Lovestone S. (2017). Alzheimer’s disease in humans and other animals: A consequence of postreproductive life span and longevity rather than aging. Alzheimer’s Dement. J. Alzheimer’s Assoc..

[17] Sarasa M., Pesini P. (2009). Natural non-trasgenic animal models for research in Alzheimer’s disease. Curr. Alzheimer Res..

[18] Page-Karjian A., Lo C.F., Ritchie B., Harms C., Rotstein D.S., Han S., Hassan S.M., Lehner A.F., Buchweitz J.P., Thayer V.G. (2020). Anthropogenic Contaminants and Histopathological Findings in Stranded Cetaceans in the Southeastern United States, 2012–2018. Front. Mar. Sci..

[19] Reif J.S., Schaefer A.M., Bossart G.D. (2015). Atlantic Bottlenose Dolphins (*Tursiops truncatus*) as A Sentinel for Exposure to Mercury in Humans: Closing the Loop. Vet. Sci..

[20] Metcalf J.S., Codd G.A. (2020). Co-Occurrence of Cyanobacteria and Cyanotoxins with Other Environmental Health Hazards: Impacts and Implications. Toxins.

[21] Rush T., Liu X., Lobner D. (2012). Synergistic toxicity of the environmental neurotoxins methylmercury and beta-N-methylamino-L-alanine. Neuroreport.

[22] Bossart G.D. (2011). Marine mammals as sentinel species for oceans and human health. Vet. Pathol..

[23] Mirra S.S., Heyman A., McKeel D., Sumi S.M., Crain B.J., Brownlee L.M., Vogel F.S., Hughes J.P., van Belle G., Berg L. (1991). The Consortium to Establish a Registry for Alzheimer’s Disease (CERAD). Part II. Standardization of the neuropathologic assessment of Alzheimer’s disease. Neurology.

[24] Field N.C., Metcalf J.S., Caller T.A., Banack S.A., Cox P.A., Stommel E.W. (2013). Linking beta-methylamino-L-alanine exposure to sporadic amyotrophic lateral sclerosis in Annapolis, MD. Toxicon Off. J. Int. Soc. Toxinol..

[25] Banack S.A., Metcalf J.S., Bradley W.G., Cox P.A. (2014). Detection of cyanobacterial neurotoxin beta-N-methylamino-l-alanine within shellfish in the diet of an ALS patient in Florida. Toxicon Off. J. Int. Soc. Toxinol..

[26] Masseret E., Banack S., Boumediene F., Abadie E., Brient L., Pernet F., Juntas-Morales R., Pageot N., Metcalf J., Cox P. (2013). Dietary BMAA exposure in an amyotrophic lateral sclerosis cluster from southern France. PLoS ONE.

[27] Banack S.A., Murch S.J., Cox P.A. (2006). Neurotoxic flying foxes as dietary items for the Chamorro people, Marianas Islands. J. Ethnopharmacol..

[28] Monson C.S., Banack S.A., Cox P.A. (2003). Conservation implications of Chamorro consumption of flying foxes as a possible cause of amyotrophic lateral sclerosis–parkinsonism dementia complex in Guam. Conserv. Biol..

[29] Finch C.E., Kulminski A.M. (2019). The Alzheimer’s Disease Exposome. Alzheimer’s Dement. J. Alzheimer’s Assoc..

[30] Eto K., Marumoto M., Takeya M. (2010). The pathology of methylmercury poisoning (Minamata disease): The 50th Anniversary of Japanese Society of Neuropathology. Neuropathol. Off. J. Jpn. Soc. Neuropathol..

[31] Cox P.A., Sacks O.W. (2002). Cycad neurotoxins, consumption of flying foxes, and ALS-PDC disease in Guam. Neurology.

[32] Bell E.A. (2009). The discovery of BMAA, and examples of biomagnification and protein incorporation involving other non-protein amino acids. Amyotroph. Lateral Scler. Off. Publ. World Fed. Neurol. Res. Group Motor Neuron Dis..

[33] Foley M.M., Seidel I., Sevier J., Wendt J., Kogan M. (2020). One man’s swordfish story: The link between Alzheimer’s disease and mercury exposure. Complement. Ther. Med..

[34] Wu J., Hilborn E.D., Schaeffer B.A., Urquhart E., Coffer M.M., Lin C.J., Egorov A.I. (2021). Acute health effects associated with satellite-determined cyanobacterial blooms in a drinking water source in Massachusetts. Environ. Health Glob. Access Sci. Source.

[35] Taylor D.L., Calabrese N.M. (2018). Mercury content of blue crabs (*Callinectes sapidus*) from southern New England coastal habitats: Contamination in an emergent fishery and risks to human consumers. Mar. Pollut. Bull..

[36] Brand L.E. (2009). Human exposure to cyanobacteria and BMAA. Amyotroph. Lateral Scler. Off. Publ. World Fed. Neurol. Res. Group Motor Neuron Dis..

[37] Hong Y.S., Kim Y.M., Lee K.E. (2012). Methylmercury exposure and health effects. J. Prev. Med. Public Health.

[38] Lance E., Arnich N., Maignien T., Bire R. (2018). Occurrence of beta-N-methylamino-l-alanine (BMAA) and Isomers in Aquatic Environments and Aquatic Food Sources for Humans. Toxins.

[39] Buckman K.L., Mason R.P., Seelen E., Taylor V.F., Balcom P.H., Chipman J., Chen C.Y. (2021). Patterns in forage fish mercury concentrations across Northeast US estuaries. Environ. Res..

[40] Xie X., Basile M., Mash D.C. (2013). Cerebral uptake and protein incorporation of cyanobacterial toxin beta-N-methylamino-L-alanine. Neuroreport.

[41] Waidyanatha S., Ryan K., Sanders J.M., McDonald J.D., Wegerski C.J., Doyle-Eisle M., Garner C.E. (2018). Disposition of beta-N-methylamino-l-alanine (L-BMAA), a neurotoxin, in rodents following a single or repeated oral exposure. Toxicol. Appl. Pharmacol..

[42] Duncan M.W., Villacreses N.E., Pearson P.G., Wyatt L., Rapoport S.I., Kopin I.J., Markey S.P., Smith Q.R. (1991). 2-amino-3-(methylamino)-propanoic acid (BMAA) pharmacokinetics and blood-brain barrier permeability in the rat. J. Pharmacol. Exp. Ther..

[43] Rand M.D., Caito S.W. (2019). Variation in the biological half-life of methylmercury in humans: Methods, measurements and meaning. Biochim. Biophys. Acta Gen. Subj..

[44] Dunlop R.A., Cox P.A., Banack S.A., Rodgers K.J. (2013). The non-protein amino acid BMAA is misincorporated into human proteins in place of l-serine causing protein misfolding and aggregation. PLoS ONE.

[45] Davis D.A., Cox P.A., Banack S.A., Lecusay P.D., Garamszegi S.P., Hagan M.J., Powell J.T., Metcalf J.S., Palmour R.M., Beierschmitt A. (2020). l-Serine Reduces Spinal Cord Pathology in a Vervet Model of Preclinical ALS/MND. J. Neuropathol. Exp. Neurol..

[46] Spencer P.S., Hugon J., Ludolph A., Nunn P.B., Ross S.M., Roy D.N., Schaumburg H.H. (1987). Discovery and partial characterization of primate motor-system toxins. Ciba Found. Symp..

[47] Spencer P.S., Nunn P.B., Hugon J., Ludolph A.C., Ross S.M., Roy D.N., Robertson R.C. (1987). Guam amyotrophic lateral sclerosis-parkinsonism-dementia linked to a plant excitant neurotoxin. Science.

[48] Cox P.A., Banack S.A., Murch S.J. (2003). Biomagnification of cyanobacterial neurotoxins and neurodegenerative disease among the Chamorro people of Guam. Proc. Natl. Acad. Sci. USA.

[49] Oyanagi K., Yamazaki M., Hashimoto T., Asakawa M., Wakabayashi K., Takahashi H. (2015). Hippocampal sclerosis in the parkinsonism-dementia complex of Guam: Quantitative examination of neurons, neurofibrillary tangles, and TDP-43 immunoreactivity in CA1. Neuropathol. Off. J. Jpn. Soc. Neuropathol..

[50] Nelson P.T., Dickson D.W., Trojanowski J.Q., Jack C.R., Boyle P.A., Arfanakis K., Rademakers R., Alafuzoff I., Attems J., Brayne C. (2019). Limbic-predominant age-related TDP-43 encephalopathy (LATE): Consensus working group report. Brain.

[51] Mackenzie I.R., Rademakers R., Neumann M. (2010). TDP-43 and FUS in amyotrophic lateral sclerosis and frontotemporal dementia. Lancet Neurol..

[52] Josephs K.A., Whitwell J.L., Knopman D.S., Hu W.T., Stroh D.A., Baker M., Rademakers R., Boeve B.F., Parisi J.E., Smith G.E. (2008). Abnormal TDP-43 immunoreactivity in AD modifies clinicopathologic and radiologic phenotype. Neurology.

[53] Geser F., Winton M.J., Kwong L.K., Xu Y., Xie S.X., Igaz L.M., Garruto R.M., Perl D.P., Galasko D., Lee V.M. (2008). Pathological TDP-43 in parkinsonism-dementia complex and amyotrophic lateral sclerosis of Guam. Acta Neuropathol..

[54] Crary J.F., Trojanowski J.Q., Schneider J.A., Abisambra J.F., Abner E.L., Alafuzoff I., Arnold S.E., Attems J., Beach T.G., Bigio E.H. (2014). Primary age-related tauopathy (PART): A common pathology associated with human aging. Acta Neuropathol..

[55] Ash P.E.A., Dhawan U., Boudeau S., Lei S., Carlomagno Y., Knobel M., Al Mohanna L.F.A., Boomhower S.R., Newland M.C., Sherr D.H. (2019). Heavy Metal Neurotoxicants Induce ALS-Linked TDP-43 Pathology. Toxicol. Sci..

[56] Munoz-Saez E., de Munck E., Arahuetes R.M., Solas M.T., Martinez A.M., Miguel B.G. (2013). beta-N-methylamino-L-alanine induces changes in both GSK3 and TDP-43 in human neuroblastoma. J. Toxicol. Sci..

[57] Scott L.L., Downing T.G. (2017). A Single Neonatal Exposure to BMAA in a Rat Model Produces Neuropathology Consistent with Neurodegenerative Diseases. Toxins.

[58] Rice K.M., Walker E.M., Wu M., Gillette C., Blough E.R. (2014). Environmental mercury and its toxic effects. J. Prev. Med. Public Health.

[59] Li X., Pan J., Wei Y., Ni L., Xu B., Deng Y., Yang T., Liu W. (2021). Mechanisms of oxidative stress in methylmercury-induced neurodevelopmental toxicity. Neurotoxicology.

[60] Yorifuji T. (2020). Lessons From an Early-stage Epidemiological Study of Minamata Disease. J. Epidemiol..

[61] U.S. Food and Drug Administration (2017). Mercury Concentrations in Fish from the FDA Monitoring Program (1990–2010).

[62] Godfrey M.E., Wojcik D.P., Krone C.A. (2003). Apolipoprotein E genotyping as a potential biomarker for mercury neurotoxicity. J. Alzheimer’s Dis. JAD.

[63] Siblerud R., Mutter J., Moore E., Naumann J., Walach H. (2019). A Hypothesis and Evidence That Mercury May be an Etiological Factor in Alzheimer’s Disease. Int. J. Environ. Res. Public. Health.

[64] Yokoo E.M., Valente J.G., Grattan L., Schmidt S.L., Platt I., Silbergeld E.K. (2003). Low level methylmercury exposure affects neuropsychological function in adults. Environ. Health Glob. Access Sci. Source.

[65] Eto K., Takizawa Y., Akagi H., Haraguchi K., Asano S., Takahata N., Tokunaga H. (1999). Differential diagnosis between organic and inorganic mercury poisoning in human cases—The pathologic point of view. Toxicol. Pathol..

[66] Bjorkman L., Lundekvam B.F., Laegreid T., Bertelsen B.I., Morild I., Lilleng P., Lind B., Palm B., Vahter M. (2007). Mercury in human brain, blood, muscle and toenails in relation to exposure: An autopsy study. Environ. Health A Glob. Access Sci. Source.

[67] Davis L.E., Kornfeld M., Mooney H.S., Fiedler K.J., Haaland K.Y., Orrison W.W., Cernichiari E., Clarkson T.W. (1994). Methylmercury poisoning: Long-term clinical, radiological, toxicological, and pathological studies of an affected family. Ann. Neurol..

[68] Rao S.D., Banack S.A., Cox P.A., Weiss J.H. (2006). BMAA selectively injures motor neurons via AMPA/kainate receptor activation. Exp. Neurol..

[69] Silva D.F., Candeias E., Esteves A.R., Magalhaes J.D., Ferreira I.L., Nunes-Costa D., Rego A.C., Empadinhas N., Cardoso S.M. (2020). Microbial BMAA elicits mitochondrial dysfunction, innate immunity activation, and Alzheimer’s disease features in cortical neurons. J. Neuroinflamm..

[70] Hiraoka H., Nakahara K., Kaneko Y., Akiyama S., Okuda K., Iwawaki T., Fujimura M., Kumagai Y., Takasugi N., Uehara T. (2017). Modulation of Unfolded Protein Response by Methylmercury. Biol. Pharm. Bull..

[71] Dunlop R.A., Powell J.T., Metcalf J.S., Guillemin G.J., Cox P.A. (2018). L-Serine-Mediated Neuroprotection Includes the Upregulation of the ER Stress Chaperone Protein Disulfide Isomerase (PDI). Neurotox. Res..

[72] Sanderson C.E., Alexander K.A. (2020). Unchartered waters: Climate change likely to intensify infectious disease outbreaks causing mass mortality events in marine mammals. Glob. Chang. Biol..

[73] Di Guardo G. (2018). Alzheimer’s disease, cellular prion protein, and dolphins. Alzheimer’s Dement. J. Alzheimer’s Assoc..

[74] Guzman-Verri C., Gonzalez-Barrientos R., Hernandez-Mora G., Morales J.A., Baquero-Calvo E., Chaves-Olarte E., Moreno E. (2012). Brucella ceti and brucellosis in cetaceans. Front. Cell. Infect. Microbiol..

[75] Davison N.J., Brownlow A., Doeschate M.T., Dale E.J., Foster G., Muchowski J., Perrett L.L., Rocchi M., Whatmore A.M., Dagleish M.P. (2021). Neurobrucellosis due to Brucella ceti ST26 in Three Sowerby’s Beaked Whales (*Mesoplodon bidens*). J. Comp. Pathol..

[76] Hernandez-Mora G., Gonzalez-Barrientos R., Morales J.A., Chaves-Olarte E., Guzman-Verri C., Barquero-Calvo E., De-Miguel M.J., Marin C.M., Blasco J.M., Moreno E. (2008). Neurobrucellosis in stranded dolphins, Costa Rica. Emerg. Infect. Dis..

[77] Geraci J.R., Lounsbury V.L., Yates N. (2005). Marine Mammals Ashore, A Field Guide for Strandings.

[78] LeDuc R.G., Perrin W.F., Dizon A.E. (1999). Phylogenetic Relationships Among the Delphinid Cetaceans Based on Full Cytochrome B Sequences. Mar. Mammal Sci..

[79] Chen I.H., Chou L.S., Chou S.J., Wang J.H., Stott J., Blanchard M., Jen I.F., Yang W.C. (2015). Selection of suitable reference genes for normalization of quantitative RT-PCR in peripheral blood samples of bottlenose dolphins *(Tursiops truncatus*). Sci. Rep..

[80] Schmittgen T.D., Livak K.J. (2008). Analyzing real-time PCR data by the comparative C(T) method. Nat. Protoc..

[81] Office of Water, USEPA (2001). Method 1630, Methyl Mercury in Water by Distillation, Aqueous Ethylation, Purge and Trap, and Cold Vapor Atomic Fluorescence Spectrometry.

[82] Oelschlager H.H., Haas-Rioth M., Fung C., Ridgway S.H., Knauth M. (2008). Morphology and evolutionary biology of the dolphin (Delphinus sp.) brain—MR imaging and conventional histology. Brain Behav. Evol..

[83] Mirra S.S., Hart M.N., Terry R.D. (1993). Making the diagnosis of Alzheimer’s disease. A primer for practicing pathologists. Arch. Pathol. Lab. Med..

[84] Hof P.R., Chanis R., Marino L. (2005). Cortical complexity in cetacean brains. Anat. Rec. Part A Discov. Mol. Cell. Evol. Biol..

