# Supplementary material for: BMAA, Methylmercury, and Mechanisms of Neurodegeneration in Dolphins: A Natural Model of Toxin Exposure
Source: Toxins (Basel). 2021 Oct 1;13(10):697. doi: 10.3390/toxins13100697 (PMC8540894; doi:10.3390/toxins13100697)
Supplement: Supplementary file 1 [file toxins-13-00697-s001.zip › Davis et al 2021 Supplementary Figure S1 Toxins .pdf]

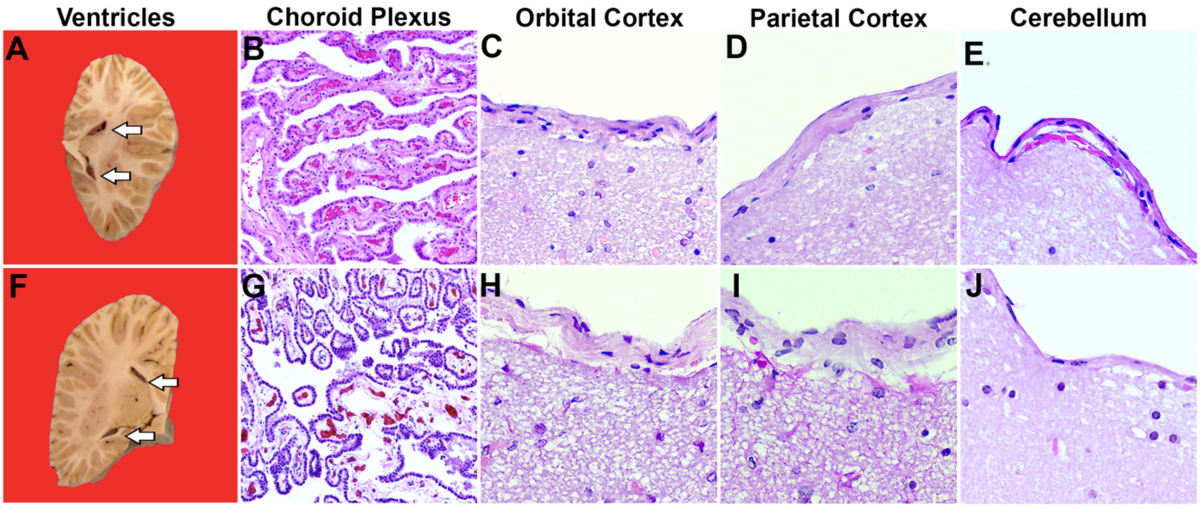

Supplementary Figure S1: Pathological Findings Associated with Neurobrucellosis Were not Observed in Our Study Cohort. Comparative grossing photos and digital pathology scans of representative tissue sections from a brucellosis^{+} dolphin (IFAW 12-228 Dd, A-E; top panels) and two brucellosis^{-} dolphins (IFAW 12-201 Dd (F, H-J) & IFAW 12-198 Dd (G); bottom panels). (A, F) Coronal sections displaying an unremarkable cerebral cortex and ventricles of normal size in both brucellosis^{+} and brucellosis^{-} dolphins (arrows). (B-E, G-j) H&E stained tissue sections of the dolphin choroid plexus, orbital cortex, parietal cortex, and cerebellum. The choroid plexus of all dolphins in this study displayed an intact ependyma without the presence of a subependymal mononuclear cell invasion. For the cerebral cortex and cerebellum, sections show normal distribution of cellular components in the leptomeninges and the absence of meningoencephalitis that is commonly associated with neurobrucellosis. Digital scans 10x (B & G); 40x (C-E, H-J)
