# Supplementary material for: BMAA, Methylmercury, and Mechanisms of Neurodegeneration in Dolphins: A Natural Model of Toxin Exposure
Source: Toxins (Basel). 2021 Oct 1;13(10):697. doi: 10.3390/toxins13100697 (PMC8540894; doi:10.3390/toxins13100697)
Supplement: Supplementary file 1 [file toxins-13-00697-s001.zip › Davis et al 2021 Supplementary Table S1 Toxins.pdf]

Supplementary Table S1: Stranded Dolphins

No.	Common Name	Species	Agency ID	Observed	Stranding Date	Stranding Location	Necropsy Findings	Sex	Age Class	Length (cm)	Weight (kg)	RIN
1	Short-Beaked Common Dolphin	*D. delphis*	IFAW 12-198 Dd	Beached	03/06/12	Power's Landing	Unknown	F	Adult	186	77	9.3
2	Short-Beaked Common Dolphin	*D. delphis*	IFAW 12-200 Dd	Beached	03/07/12	Corporation Beach	Unknown	M	Subadult	157	42	9.8
3	Short-Beaked Common Dolphin	*D. delphis*	IFAW 12-201 Dd	Beached	03/09/12	Skaget Beach	Unknown	F	Adult	206	103	9.7
4	Short-Beaked Common Dolphin	*D. delphis*	IFAW 12-205 Dd	Beached	03/11/12	Duck Creek	Unknown	F	Subadult	167	57	9.2
5	Short-Beaked Common Dolphin	*D. delphis*	IFAW 12-223 Dd	Beached	03/12/12	Skaget Beach	Brucellosis	M	Subadult	185	*N/A*	9.6
6	Short-Beaked Common Dolphin	*D. delphis*	IFAW 12-228 Dd	Beached	04/05/12	Black Fish Creek	Brucellosis	M	Adult	211	86	9.2
7	Short-Beaked Common Dolphin	*D. delphis*	IFAW 12-229 Dd	Swimming	04/03/12	Black Fish Creek	Brucellosis	M	Subadult	181	*N/A*	9.9

cm: centimeters; **F:** female; **kg:** kilograms; **M:** male; ***N/A*:** data not available; **RIN:** RNA integrity number;
