# Supplementary material for: BMAA, Methylmercury, and Mechanisms of Neurodegeneration in Dolphins: A Natural Model of Toxin Exposure
Source: Toxins (Basel). 2021 Oct 1;13(10):697. doi: 10.3390/toxins13100697 (PMC8540894; doi:10.3390/toxins13100697)
Supplement: Supplementary file 1 [file toxins-13-00697-s001.zip › Davis et al 2021 Supplementary Table S2 Toxins.pdf]

Supplementary Table S2: Dolphin qPCR primers

Gene Symbol	Name	Assay ID	Protein in Dolphins	Protein in Humans	Associated Disease in Humans	Assay Name	SeqAC	Transcript variant	Amplicon Length	Location
APP	Amyloid Beta Precursor Protein	APGZHYX	Amyloid-beta A4 protein	Amyloid Beta Precursor Protein	AD	APP_1	XM_019922563.1	X1	64	550
		APGZHYX				APP_1	XM_019922564.1	X2	64	550
C9orf72	Chromosome 9 Open Reading Frame 72	APKA64T	Guanine Nucleotide Exchange C9orf72 Homolog Isoform X1, X2	Guanine nucleotide exchange C9orf72	ALS; FTD	C9ORF72_1	XM_004318999.2	---	61	817
GRN	Granulin Precursor	APRWHFG	Progranulin Isoform X1, X2	Progranulin	AD; FTD	GRN_1	XM_019939325.1	----	64	158
MAPT	Microtubule Associated Protein Tau	APU66KC	Microtubule-Associated Protein Tau	Microtubule-Associated Protein Tau	AD; ALS/PDC; FTD; PART	MAPT_1	XM_019944330.1	X1	59	397
		APU66KC				MAPT_1	XM_019944331.1	X2	59	397
		APU66KC				MAPT_1	XM_019944332.1	X3	59	310
		APU66KC				MAPT_1	XM_019944333.1	X4	59	397
		APU66KC				MAPT_1	XM_019944334.1	X5	59	397
		APU66KC				MAPT_1	XM_019944335.1	X6	59	310
		APU66KC				MAPT_1	XM_019944336.1	X7	59	397
		APU66KC				MAPT_1	XM_019944337.1	X8	59	310
		APU66KC				MAPT_1	XM_019944338.1	X9	59	110
		APU66KC				MAPT_1	XM_019944339.1	X10	59	110
PSEN1	Presenilin 1	APYMNA3	Presenilin	Presenilin-1	AD	PSEN1_2	XM_019921707.1	X1	67	1855
		APYMNA3				PSEN1_2	XM_019921714.1	X2	67	1843
PSEN2	Presenilin 2	AP2XCFX	Presenilin	Presenilin-2	AD	PSEN2_2	XM_019920287.1	---	55	1812
TARDBP	TAR DNA Binding Protein	AP7DT6P	TAR DNA-binding protein 43	TAR DNA-binding protein 43	AD, ALS, FTD, LATE	TARDBP_1	XM_019930884.1	X1	67	822
		AP7DT6P				TARDBP_1	XM_019930886.1	X3	67	801
RPS9	40S Ribosomal Protein S9	AP324ZV	40S ribosomal protein S9	40S ribosomal protein S9	House Keeping Gene	RPS9_1	EU638309.1	partial cds	55	67
		AP324ZV				RPS9_1	XM_019927380.1	X1	55	207
		AP324ZV				RPS9_1	XM_004332149.2	X2	55	124
		AP324ZV				RPS9_1	XM_019927381.1	X3	55	329
