# Supplementary material for: BMAA, Methylmercury, and Mechanisms of Neurodegeneration in Dolphins: A Natural Model of Toxin Exposure
Source: Toxins (Basel). 2021 Oct 1;13(10):697. doi: 10.3390/toxins13100697 (PMC8540894; doi:10.3390/toxins13100697)
Supplement: Supplementary file 1 [file toxins-13-00697-s001.zip › Davis et al 2021 Supplementary Table S3 Toxins .pdf]

**Supplementary Table S3**: Gene transcription Fold Change in Stranded Dolphins

| *PL* | *APP* | *PSEN 1* | *PSEN 2* | *MAPT* | *GRN* | *TARDBP* | *C9orf72* |
|---|---|---|---|---|---|---|---|
| [⊿]IFAW 12-228 Dd | 0.91 | 1.02 | 0.99 | 1.04 | 1.03 | 0.99 | 1.05 |
| IFAW 12-223 Dd | 0.95 | 1.50 | 0.96 | 1.44 | 1.05 | 1.77 | 1.73 |
| IFAW 12-200 Dd | 0.74 | 0.78 | 0.98 | 0.96 | 0.74 | 0.71 | 0.67 |
| IFAW 12-198 Dd | 1.10 | 1.79 | 1.22 | 1.27 | 0.77 | 2.84 | 2.67 |
| IFAW 12-229 Dd | 1.23 | 1.56 | 1.21 | 1.92 | 1.01 | 1.64 | 1.47 |
| IFAW 12-205 Dd | 0.84 | 1.07 | 0.89 | 1.28 | 1.00 | 1.68 | 1.51 |
| IFAW 12-201 Dd | 0.71 | 0.86 | 0.89 | 1.10 | 1.02 | 1.11 | 1.08 |
| **Median (IQR)** | **0.91 (0.36)** | **1.07 (0.70)** | **0.98 (0.32)** | **1.27 (0.40)** | **1.01 (0.26)** | **1.64 (0.78)** | **1.47 (0.68)** |
| **Min - Max** | **0.71 – 1.23** | **0.78 – 1.79** | **0.89 – 1.22** | **0.96 – 1.92** | **0.74 – 1.05** | **0.71 – 2.84** | **0.67 – 2.67** |

| *OrL* | *APP* | *PSEN 1* | *PSEN 2* | *MAPT* | *GRN* | *TARDBP* | *C9orf72* |
|---|---|---|---|---|---|---|---|
| [⊿]IFAW 12-228 Dd | 0.95 | 1.01 | 0.98 | 1.04 | 1.04 | 0.98 | 1.03 |
| IFAW 12-223 Dd | 1.28 | 1.31 | 1.09 | 1.79 | 1.25 | 2.08 | 1.70 |
| IFAW 12-200 Dd | 1.43 | 1.04 | 1.24 | 1.84 | 1.01 | 1.34 | 1.33 |
| IFAW 12-198 Dd | 1.14 | 1.18 | 1.09 | 1.42 | 0.84 | 1.91 | 1.69 |
| IFAW 12-229 Dd | 1.52 | 1.40 | 1.17 | 2.63 | 1.25 | 1.96 | 1.45 |
| IFAW 12-205 Dd | 1.38 | 1.27 | 0.96 | 2.24 | 1.60 | 2.53 | 2.17 |
| IFAW 12-201 Dd | 1.12 | 1.26 | 0.94 | 1.16 | 1.31 | 2.01 | 1.70 |
| **Median (IQR)** | **1.28 (0.31) *** | **1.26 (0.27)** | **1.09 (0.21)** | **1.79 (1.08) *** | **1.25 (0.30)** | **1.96 (0.74)** | **1.69 (0.37)** |
| **Min - Max** | **0.95 – 1.52** | **1.01 – 1.40** | **0.94 – 1.24** | **1.04 – 2.63** | **0.84 – 1.60** | **0.98 – 2.53** | **1.03 – 2.17** |

| *Cer* | *APP* | *PSEN 1* | *PSEN 2* | *MAPT* | *GRN* | *TARDBP* | *C9orf72* |
|---|---|---|---|---|---|---|---|
| [⊿]IFAW 12-228 Dd | 0.94 | 0.98 | 0.98 | 0.98 | 0.99 | 0.99 | 0.95 |
| IFAW 12-223 Dd | 0.92 | 1.32 | 0.85 | 1.14 | 0.94 | 1.99 | 1.03 |
| IFAW 12-200 Dd | 1.13 | 1.21 | 1.03 | 1.12 | 0.88 | 1.57 | 1.34 |
| IFAW 12-198 Dd | 1.31 | 2.94 | 1.21 | 1.14 | 1.16 | 2.92 | 2.69 |
| IFAW 12-229 Dd | 1.03 | 1.01 | 1.13 | 1.46 | 1.00 | 1.41 | 1.03 |
| IFAW 12-205 Dd | 1.00 | 1.22 | 0.79 | 1.20 | 1.03 | 2.13 | 2.01 |
| IFAW 12-201 Dd | 1.01 | 1.08 | 0.90 | 1.69 | 0.95 | 1.93 | 1.91 |
| **Median (IQR)** | **1.01 (0.19)** | **1.21 (0.31)** | **0.98 (0.28)** | **1.14 (0.34)** | **0.99 (0.09)** | **1.93 (0.72)** | **1.34 (0.98)** |
| **Min - Max** | **0.92 – 1.31** | **0.98 – 2.94** | **0.79 – 1.21** | **0.98 – 1.69** | **0.88 – 1.16** | **0.99 – 2.92** | **0.95 – 2.69** |

***Cer***: cerebellum; ***PL***: parietal Lobe; ***OrL***: orbital Lobe; **[⊿]:** Dolphin used for normalization of qPCR data; *$P<0.05$ ANOVA
